# The Predictive Role of Serum Triglyceride to High-Density Lipoprotein Cholesterol Ratio According to Renal Function in Patients with Acute Myocardial Infarction

**DOI:** 10.1371/journal.pone.0165484

**Published:** 2016-10-27

**Authors:** Jin Sug Kim, Weon Kim, Jong Shin Woo, Tae Won Lee, Chun Gyoo Ihm, Yang Gyoon Kim, Joo Young Moon, Sang Ho Lee, Myung Ho Jeong, Kyung Hwan Jeong, Korea Acute Myocardial Infarction Registry Investigators

**Affiliations:** 1 Department of Medicine, Graduate School, Kyung Hee University, Seoul, Republic of Korea; 2 Division of Cardiology, Department of Internal Medicine, School of Medicine, Kyung Hee University, Seoul, Republic of Korea; 3 Division of Nephrology, Department of Internal Medicine, School of Medicine, Kyung Hee University, Seoul, Republic of Korea; 4 Division of Cardiology, Department of Medicine, Chonnam National University Hospital, Gwangju, Republic of Korea; University of Kansas Medical Center, UNITED STATES

## Abstract

**Objective:**

A high serum triglyceride to high-density lipoprotein cholesterol (TG/HDL-C) ratio has been reported as an independent predictor for cardiovascular events in the general population. However, the prognostic value of this ratio in patients with renal dysfunction is unclear. We examined the association of the TG/HDL-C ratio with major adverse cardiovascular events (MACEs) according to renal function in patients with acute myocardial infarction (AMI).

**Method:**

This study was based on the Korea Acute Myocardial Infarction Registry database. Of 13,897 patients who were diagnosed with AMI, the study population included the 7,016 patients with available TG/HDL-C ratio data. Patients were stratified into three groups according to their estimated glomerular filtration rate (eGFR), and the TG/HDL-C ratio was categorized into tertiles. We investigated 12-month MACEs, which included cardiac death, myocardial infarction, and repeated percutaneous coronary intervention or coronary artery bypass grafting.

**Results:**

During the 12-month follow up period, 593 patients experienced MACEs. There was a significant association between the TG/HDL-C ratio and MACEs (p<0.001) in the entire study cohort. Having a TG/HDL-C ratio value in the highest tertile of TG/HDL-C ratio was an independent factor associated with increased risk of MACEs (hazard ratio [HR], 1.56; 95% confidence interval [CI], 1.26–1.93; p<0.001). Then we performed subgroup analyses according to renal function. In patients with normal renal function (eGFR ≥ 90 ml/min/1.73m^2^) and mild renal dysfunction (eGFR ≥ 60 to < 90ml/min/1.73m^2^), a higher TG/HDL-C ratio was significantly associated with increased risk of MACEs (HR, 1.64; 95% CI, 1.04–2.60; p = 0.035; and HR, 1.56; 95% CI, 1.14–2.12; p = 0.005, respectively). However, in patients with moderate renal dysfunction (eGFR < 60 ml/min/1.73m^2^), TG/HDL-C ratio lost its predictive value on the risk of MACEs (HR, 1.23; 95% CI, 0.82–1.83; p = 0.317).

**Conclusions:**

In patients with AMI, TG/HDL-C ratio is a useful independent predictor of 12-month MACEs. However, this ratio does not have predictive power in patients with moderate renal dysfunction.

## Introduction

Cardiovascular disease (CVD) is the leading cause of global mortality and a growing worldwide public health problem [1]. Since prediction of CVD risk has an important role in CVD prevention, many attempts have been made to refine and improve the risk assessment criteria [2]. Though traditional risk factors such as age, systolic blood pressure (SBP), smoking status, and total cholesterol level are well established predictors of CVD risk, these factors do not fully account for the increased CVD risk [2, 3]. Recently some parameters associated with inflammation, oxidative stress, insulin resistance, and endothelial dysfunction have been reported that are better at risk stratification in CVD [4]. The serum triglyceride to high-density lipoprotein cholesterol (TG/HDL-C) ratio, which is known as an atherogenic index of plasma, is one such parameter [5]. The serum TG/HDL-C ratio is considered to be a better indicator of insulin resistance and CVD risk than are other lipid profiles [6]. The predictive role of the TG/HDL-C ratio on CVD risk has been analyzed in patients with several disorders such as coronary heart disease, diabetes mellitus (DM), and hypertension [3, 7–9]. Although accumulating evidence supports the predictive power of the TG/HDL-C ratio in general and in certain subgroups, very few studies analyzed this in patients with renal dysfunction [4, 10]. The TG/HDL-C ratio has also not been included in several studies that examined the association between dyslipidemia and CVD risk in patients with renal impairment.

The aim of this study was to investigate the predictive role of the TG/HDL-C ratio according to renal function. This is an observational study that uses a nationwide registry to determine whether the TG/HDL-C ratio could predicts major adverse cardiovascular events (MACEs) in patients with acute myocardial infarction (AMI) who were stratified according to renal function.

## Materials and Methods

### Study design and population

**Fig 1:**
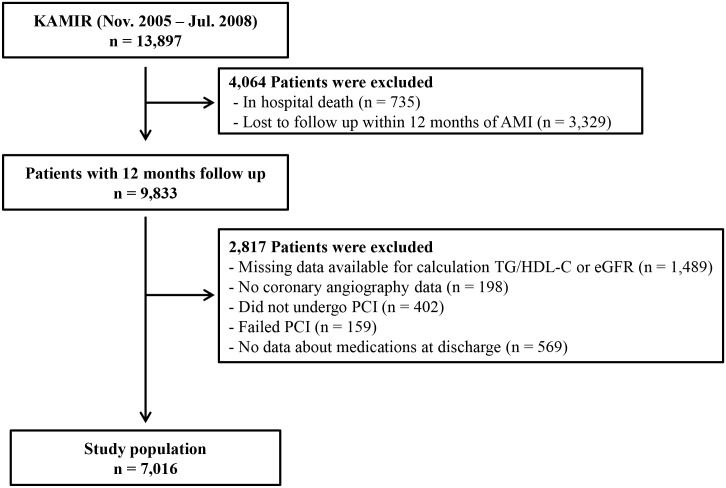
Study flow chart. KAMIR, Korea Acute Myocardial Infarction Registry; AMI, acute myocardial infarction; TG/HDL-C, triglyceride to high-density lipoprotein cholesterol; eGFR, estimated glomerular filtration rate; PCI, percutaneous coronary intervention.

The study population was derived from the Korea Acute Myocardial Infarction Registry (KAMIR) database. The KAMIR is a prospective, multicenter, on-line registry of AMI patients in Korea that is been supported by the Korean Society of Cardiology since November 2005. From November 2005 to July 2008, 13,897 patients diagnosed with AMI were enrolled in the KAMIR database. We excluded patients with in-hospital death or who were lost to follow-up within 12 months of AMI. Also, patients who lacked data necessary for the calculation of the TG/HDL-C ratio or estimated glomerular filtration rate (eGFR) were excluded, as were patients with no data on coronary angiography or failed percutaneous coronary intervention (PCI). Additionally, patients missing data about medications were not included. Overall, as shown in Fig 1, a total of 7,016 patients were enrolled in this study. Because the TG/HDL-C ratio was not normally distributed in the study population, we stratified the patients into tertiles according to their TG/HDL-C ratio: low (< 1.84, n = 2339); middle (≥ 1.84 to < 3.35, n = 2,338); and high (≥ 3.35, n = 2,339).

After investigating the predictive value of the TG/HDL-C ratio in the entire study population, we then analyzed the population separately according to renal function: normal renal function group (n = 2,021); mild renal dysfunction group (n = 3,581); and moderate renal dysfunction group (n = 1,414). All study procedures complied with the ethical guidelines of the 1975 Declaration of Helsinki, as revised in 2000. The study protocol was approved by the institutional review board of all centers, and the approval number was 05–49 of Chonnam National University Hospital. The participating sites are listed in an appendix. Given the retrospective design of the project, this institutional review board waived the need for consent. The format recommended by the Strengthening the Reporting of Observational Studies in Epidemiology (STROBE) guidelines[11] were used in conducting the study.

### Variables and definitions

Baseline variables included age, gender, body mass index (BMI), and several cardiovascular risk factors such as hypertension, DM, dyslipidemia, current smoking, and previous coronary artery disease (CAD). Laboratory findings including lipid profile and cardiac markers, and medications prescribed at discharge were recorded. Renal function was defined by eGFR. The eGFR was calculated using the Modification of Diet in Renal Disease (MDRD) study equation: 186.3 × (serum creatinine^-1.0154^) × (age^-0.203^) × 0.742 (if female) [12]. Normal renal function, mild renal dysfunction, and moderate renal dysfunction were defined as eGFR ≥ 90 mL/min/1.73m^2^, ≥ 60 to < 90 mL/min/1.73m^2^, and < 60 mL/min/1.73m^2^, respectively. The TG/HDL-C ratio was calculated as TG (mg/dL) divided by HDL-C (mg/dL). For both whole study population analysis and subgroup analyses, patients were divided into three groups according to TG/HDL-C ratio tertile.

### Study outcomes

The primary outcome of this study was 12-month MACEs, including cardiac death, recurrent MI, and repeated percutaneous coronary intervention (target lesion or target vessel revascularization, or non-target vessel revascularization) or coronary artery bypass grafting (CABG). Cardiac death was defined as death from arrhythmic causes or mechanical complications including free wall rupture and ventricular septal rupture. Recurrent MI was defined as recurrent clinical features with new electrocardiographic findings that were compatible with MI, or increased levels of biochemical markers.

### Statistical analysis

All statistical analyses were performed using SPSS software version 19.0 (SPSS Inc., Chicago, IL, USA). A p value < 0.05 was considered to be statistically significant. Presence of the normal distribution was evaluated using the Kolmogorov-Smirnov test. Continuous variables are presented as means ± standard deviations (SDs), and categorical data are reported as frequencies and percentages. The Chi-squared test, Kruskal-Wallis test, and one-way ANOVA were used to compare the baseline characteristics of patients classified by tertile of TG/HDL-C ratio or renal function. Survival curves were used to investigate the effect of TG/HDL-C ratio tertile on 12-month MACEs. Multiple Cox proportional regression analysis was used to investigate the independent association of the TG/HDL-C ratio with MACEs after adjustment for several confounders. The TG/HDL-C ratio and eGFR were included as categorical variables in these models. The fully adjusted Cox proportional regression model was adjusted for age, gender, body mass index (BMI), HTN, DM, smoking, and previous CAD. The results are presented as hazard ratios (HRs) ± 95% confidence intervals (CI) and statistical significance is indicated.

## Results

### Baseline characteristics

A total of 7,016 patients who were diagnosed with AMI were included in this study. Baseline characteristics of the study population across tertiles of TG/HDL-C ratio are summarized in Table 1. The average age of the study subjects was 62.3 ± 12.3 years, and 73.3% were male. The frequencies of hypertension, DM, and dyslipidemia were 47%, 26.5% and 10.4%, respectively. There was a stepwise relationship between the TG/HDL-C ratio and cardiovascular risk factors. Patients with TG/HDL-C ratio value in the highest tertile more often had hypertension, DM, and dyslipidemia. There were some significant differences between the TG/HDL-C ratio subgroups in terms of medications they were prescribed at discharge.

**Table 1:** Baseline characteristics of the study subjects according to TG/HDL-C ratio tertile.

			TG/HDL-C ratio			
		Overall (n = 7,016)	Low tertile < 1.84 (n = 2,339)	Middle tertile ≥ 1.84 to < 3.35 (n = 2,338)	High tertile ≥ 3.35 (n = 2,339)	P value^a^
	Age	62.3 ± 12.3	64.9 ± 11.9	62.9 ± 11.9	59.0 ± 12.2	< 0.001
	Gender (male)	5143 (73.3%)	1637 (70.0%)	1712 (73.2%)	1794 (76.7%)	< 0.001
	Body mass index (Kg/m^2^)	24.2 ± 3.4	23.3 ± 3.4	24.2 ± 3.2	24.9 ± 3.3	< 0.001
	Systolic blood pressure (mmHg)	129.7 ± 27.5	128.5 ± 27.4	130.1 ± 27.6	130.4 ± 27.6	0.042
	Diastolic blood pressure (mmHg)	79.2 ± 16.4	78.2 ± 16.3	79.4 ± 16.4	79.9 ± 16.5	0.001
	Heart rate (bpm)	76.3 ± 17.5	76.5 ± 17.9	75.8 ± 17.4	76.7 ± 17.2	0.210
	Killip class (more than II)	660 (9.6%)	267 (11.6%)	207 (9.1%)	186 (8.2%)	<0.001
	Type of MI (STEMI:NSTEMI)		1616: 723	1478: 860	1426: 913	<0.001
Cardiovascular risk factors						
	Hypertension	3297 (47.1%)	1001 (43.0%)	1143 (48.9%)	1153 (49.5%)	<0.001
	DM	1837 (26.3%)	512 (21.9%)	607 (26.0%)	718 (30.9%)	<0.001
	Dyslipidemia	723 (10.4%)	172 (7.4%)	242 (10.4%)	309(13.3%)	<0.001
	Current smoking	4271 (61.3%)	1301 (56.0%)	1404 (60.4%)	1566 (67.5%)	<0.001
	Previous CAD	947 (13.5%)	338 (14.5%)	292 (12.5%)	317 (13.6%)	0.140
Laboratory findings						
	Ejection fraction	52.4 ± 11.7	51.4 ± 11.8	52.5 ± 11.6	53.1 ± 11.6	<0.001
	Glucose (mg/dL)	165.7 ± 70.9	160.9 ± 67.6	163.5 ± 69.0	172.7 ± 75.2	<0.001
	eGFR (mL/min/1.73m^2^)	78.7 ± 24.8	79.5 ± 24.2	78.4 ± 24.6	78.0 ± 25.7	0.091
	Max CK-MB (mg/dL)	158.3 ± 336.9	172.3 ± 387.3	153.9 ± 320.3	148.6 ± 296.3	0.041
	Max cTnI (mg/dL)	49.1 ± 86.2	49.4 ± 82.6	49.7 ± 87.2	48.1 ± 88.7	0.840
	Total cholesterol (mg/dL)	184.5 ± 42.9	175.9 ± 39.9	183.7 ± 41.6	193.7 ± 45.0	<0.001
	HDL-cholesterol (mg/dL)	44.2 ± 10.9	50.8 ± 10.9	43.9 ± 8.9	37.8 ± 8.4	<0.001
	Triglyceride (mg/dL)	124.5 ± 75.1	62.1 ± 20.4	110.0 ± 26.7	201.4 ± 75.9	0.055
	LDL-cholesterol (mg/dL)	118.8 ± 39.9	111.8 ± 35.7	120.6 ± 37.7	123.9 ± 44.6	<0.001
	hsCRP (mg/dL)	11.5 ± 51.2	8.4 ± 40.9	11.9 ± 49.9	14.1 ± 60.7	0.002
	NT-proBNP (ng/dL)	1873.2 ± 4694.8	1963.8 ± 4607.8	2030.1 ± 4838.4	1626.0 ± 4628.2	0.042
Discharge medications						
	Aspirin	6930 (97.5%)	2313 (98.9%)	2309 (98.8%)	2308 (98.7%)	0.799
	Clopidogrel	6866 (97.9%)	2281 (97.5%)	2288 (97.9%)	2297 (98.2%)	0.270
	Calcium-channel blocker	597 (8.5%)	200 (8.6%)	210 (9.0%)	187 (8.0%)	0.479
	Beta-blocker	5284 (75.3%)	1706 (72.9%)	1771 (75.7%)	1807 (77.3%)	0.002
	ACE inhibitor	4851 (69.1%)	1625 (69.5%)	1630 (69.7%)	1596 (68.2%)	0.500
	Angiotensin-receptor blocker	1076 (15.3%)	344 (14.7%)	365 (15.7%)	367 (15.7%)	0.584
	Statin	5454 (77.9%)	1770 (75.9%)	1830 (78.4%)0	1854 (79.4%)	0.013

TG/HDL-C, triglyceride to high-density lipoprotein cholesterol; DM, diabetes mellitus; MI, myocardial infarction; STEMI, ST-segment elevation myocardial infarction; NSTEMI, non ST-segment elevation myocardial infarction; CAD, coronary artery disease; eGFR, estimated glomerular filtration rate; CK-MB, cratine kinase MB; cTnI, cardiac troponin I; HDL, high-density lipoprotein; LDL, low-density lipoprotein; hsCRP, high-sensitivity C-reaction protein; NT-proBNP, N-terminal prohormone of brain natriuretic peptide

^a^Comparison between patients with low, middle, and high tertile of TG/HDL-C ratio.

### Outcomes according to the TG/HDL-C ratio tertiles

**Fig 2:**
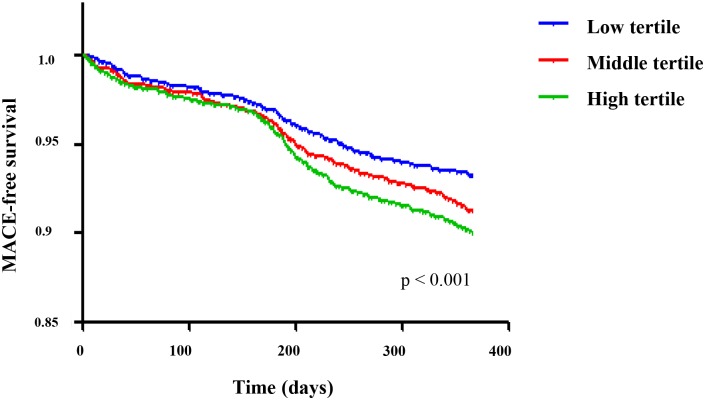
12-month MACE-free survival according to TG/HDL-C ratio tertile. MACEs, major adverse cardiovascular events; TG/HDL-C, triglyceride to high-density lipoprotein cholesterol;

Table 2 shows the prevalence of 12-month MACEs according to the TG/HDL-C ratio tertile. During the follow-up period, 593 (8.5%) patients experienced MACEs. Of the 593 patients, 100 (1.4%) had cardiogenic death, 45 (0.6%) had recurrent MI, 433 (6.2%) had repeated PCI, and 15 (0.2%) had CABG. Patients with TG/HDL-C ratio values in the higher tertiles were more likely to experience MACEs. Fig 2 shows 12-month MACE-free survival according to the TG/HDL-C ratio tertiles. The log-rank test identified that there was a significant association between TG/HDL-C tertiles and MACEs-free survival (p < 0.001). The HRs of 12-month MACEs were significant both in the middle (HR, 1.31; 95% CI, 1.06–1.61; p = 0.011) and high tertile (HR, 1.49; 95% CI, 1.22–1.83; p < 0.001). Cox regression analysis showed that being in the middle and high TG/HDL-C ratio tertiles were independent factors associated with a higher prevalence of 12-month MACEs (HR, 1.351; 95% CI, 1.092–1.672; p = 0.006; and HR, 1.564; 95% CI, 1.264–1.935; p < 0.001, respectively), along with age, gender, heart rate, Killip class, DM, glucose level, and eGFR (Table 3).

**Table 2:** Overall clinical outcomes according to TG/HDL-C ratio tertile.

		TG/HDL-C ratio			
	Overall (n = 7,016)	Low tertile < 1.84 (n = 2,339)	Middle tertile ≥ 1.84 to < 3.35 (n = 2,338)	High tertile ≥ 3.35 (n = 2,339)	P value^a^
Overall MACEs	593 (8.5%)	157 (6.7%)	204 (8.7%)	232 (9.9%)	<0.001
Cardiac death	100 (1.4%)	39 (1.7%)	33 (1.4%)	28 (1.2%)	0.397
Recurrent MI	45 (0.6%)	12 (0.5%)	11 (0.5%)	22 (0.9%)	0.084
Repeated PCI	433 (6.2%)	101 (4.3%)	155 (6.6%)	177 (7.6%)	<0.001
CABG	15 (0.2%)	5 (0.2%)	5 (0.2%)	5 (0.2%)	1.000

TG/HDL-C, triglyceride to high-density lipoprotein cholesterol; MACEs, major adverse cardiovascular events; MI, myocardial infarction; PCI: percutaneous coronary intervention; CABG, coronary artery bypass grafting

^a^Comparison between patients with low, middle, and high tertile of TG/HDL-C ratio.

**Table 3:** Predictors of MACEs in univariate and multivariate Cox regression analyses.

	Univariable		Multivariable	
	HR (95% CI)	p value	HR (95% CI)	p value
Age	1.009 (1.003–1.016)	0.007	1.005 (0.998–1.013)	0.174
Gender (male)	0.809 (0.679–0.962)	0.017	0.915 (0.753–1.111)	0.369
Body mass index (Kg/m^2^)	0.997 (0.972–1.021)	0.785		
Systolic blood pressure (mmHg)	1.001 (0.998–1.004)	0.559		
Heart rate (bpm)	1.009 (1.004–1.013)	<0.001	1.006 (1.002–-1.010)	0.008
Killip class (more than II)	1.882 (1.508–2.349)	< 0.001	1.508 (1.183–1.922)	0.001
Previous CAD	1.189 (0.952-–1.485)	0.127		
Hypertension	1.097 (0.933–1.288)	0.262		
DM	1.483 (1.251–1.758)	< 0.001	1.172 (0.956–-1.437)	0.128
Dyslipidemia	0.981 (0.751–1.281)	0.887		
Glucose (mg/dL)	1.003 (1.002–1.004)	< 0.001	1.001 (1.000–1.002)	0.035
eGFR (mL/min/1.73m^2^)	0.991 (0.988–0.994)	< 0.001	0.995 (0.992–0.999)	0.007
Total cholesterol (mg/dL)	1.000 (0.998–1.002)	0.743		
Triglyceride (mg/dL)	1.001 (1.000–1.002)	0.189		
HDL-cholesterol (mg/dL)	0.997 (0.990–1.005)	0.493		
LDL-cholesterol (mg/dL)	1.001 (0.999–1.003)	0.509		
ACE inhibitor	0.875 (0.74–1.04)	0.124		
Statin	1.070 (0.679–1.304)	0.500		
TG/HDL-C Tertile 2 vs.1	1.311 (1.065–1.614)	0.011	1.351 (1.092–1.672)	0.006
TG/HDL-C Tertile 3 vs. 1	1.499 (1.224–1.836)	< 0.001	1.564 (1.264–1.935)	< 0.001

MACEs, major adverse cardiovascular events; TG/HDL-C, triglyceride to high-density lipoprotein cholesterol; DM, diabetes mellitus; CAD, coronary artery disease; eGFR, estimated glomerular filtration rate; HDL, high-density lipoprotein; LDL, low-density lipoprotein

As shown in Table 1, about 69% of patients were prescribed ACE inhibitors as a discharge medication. Several studies have shown a relationship between ACE inhibitors and the lipid profile, especially the atherogenic index [13]. We then, analyzed the groups of patients separately according to ACE inhibitor usage to investigate whether these drugs had an impac on MACEs. Cox regression analysis showed that ACE inhibitor usage was not significantly associated with a higher prevalence of 12-month MACEs in this study population (HR, 0.875; 95% CI, 0.74–1.04; p = 0.124) (Table 3).

### Analysis of subgroups stratified according to renal function

We then, performed subgroup analyses to determine the prognostic value of the TG/HDL-C ratio according to renal function. Patients were classified into three categories by their eGFR: 2,021 (28.8%) with eGFR ≥ 90 mL/min/1.73m^2^; 3,581 (51.0%) with eGFR ≥ 60 to < 90 mL/min/1.73m^2^; and 1,414 (20.1%) with eGFR < 60 mL/min/1.73m^2^. Table 4 shows a Comparison of the baseline characteristics between the groups. Patients with lower eGFR were likely to be older and to have a higher prevalence of hypertension, DM, current smoking, and previous CAD. There was a stepwise relationship between renal function and levels of hsCRP and proBNP. Patients with lower eGFR had higher levels of hsCRP and proBNP.

**Table 4:** Baseline characteristics of the study subjects according to renal function.

			eGFR (mL/min/1.73m^2^)			
		Overall (n = 7,016)	Normal renal function ≥ 90 (n = 2,021)	Mild renal dysfuction ≥ 60 to < 90 (n = 3,581)	Moderate renal dysfunction < 60 (n = 1,414)	P value^a^
	Age	62.2±12.3	56.8±11.3	62.7±11.9	68.9±10.5	<0.001
	Gender (male)	5143 (73.3%)	1647 (81.5%)	2681 (74.9%)	815 (57.6%)	<0.001
	Body mass index (Kg/m^2^)	24.2±3.4	24.2±3.7	24.2±3.1	23.9±3.4	0.014
	Systolic blood pressure (mmHg)	129.7±27.5	131.7±25.3	129.8±26.7	126.3±31.9	<0.001
	Diastolic blood pressure (mmHg)	79.2±16.4	80.8±15.4	79.3±16.1	76.2±18.3	<0.001
	Heart rate (bpm)	76.3±17.5	76.9±15.2	75.6±16.9	77.3±21.4	0.001
	Killip class (more than II)	660 (9.6%)	106 (5.4%)	282 (8.1%)	272 (19.6%)	<0.001
	Type of MI (STEMI:NSTEMI)		1304: 717	2332: 1249	884: 530	0.222
Cardiovascular risk factors						
	Hypertension	3297 (47.1%)	757 (37.5%)	1600 (44.9%)	940 (66.7%)	<0.001
	DM	1837 (26.3%)	450 (22.3%)	819 (22.9%)	268 (40.3%)	<0.001
	Dyslipidemia	723 (10.4%)	221 (11.0%)	342 (9.6%)	160 (11.4%)	0.106
	Current smoking	4271 (61.3%)	1399 (69.5%)	223 (62.8%)	641 (45.7%)	<0.001
	Previous CAD	947 (13.5%)	188 (9.3%)	491 (13.8%)	268 (19.0%)	<0.001
Laboratory findings						
	Ejection fraction	52.3±11.7	53.4±10.9	52.6±11.6	50.2±12.6	<0.001
	Glucose (mg/dL)	165.7±70.9	156.7±62.8	161.6±66.1	189.0±86.8	<0.001
	Max CK-MB (mg/dL)	158.3±336.9	156.7±351.0	167.5±359.9	137.1±242.0	0.016
	Max cTnI (mg/dL)	49.1±86.1	51.5±86.2	47.7±53.4	48.7±92.3	0.362
	Total cholesterol (mg/dL)	184.5±42.9	186.3±42.0	185.2±41.9	179.9±45.9	<0.001
	HDL-cholesterol (mg/dL)	44.2±10.9	44.8±11.1	44.4±10.5	42.8±11.2	<0.001
	Triglyceride (mg/dL)	124.5±75.0	128.2±76.8	122.5±74.7	124.5±73.3	0.023
	TG/HDL-C ratio	3.08±2.23	3.14±2.21	2.99±2.19	3.23±2.36	0.004
	LDL-cholesterol (mg/dL)	118.8±39.9	122.5±38.1	118.9±40.2	113.5±40.8	<0.001
	hsCRP (mg/dL)	11.5±51.2	9.5±44.9	11.2±51.3	15.2±58.8	0.012
	NT-proBNP (ng/dL)	1873.2±4694.8	757.4±1480.5	1174.0±2475.4	5237.8±8741.8	<0.001
Discharge medications						
	Aspirin	6930 (98.8%)	2001 (99.0%)	3548 (99.1%)	1381 (97.7%)	<0.001
	Clopidogrel	6866 (97.9%)	1987 (98.3%)	3500 (97.7%)	1379 (97.5%)	0.219
	Calcium-channel blocker	597 (8.5%)	134 (6.6%)	304 (8.5%)	159 (11.2%)	<0.001
	Beta-blocker	5284 (75.3%)	1605 (79.4%)	2661 (74.3%)	1018 (72.0%)	<0.001
	ACE inhibitor	4851 (69.1%)	1509 (74.7%)	2479 (69.2%)	863 (61.0%)	<0.001
	Angiotensin-receptor blocker	1076 (15.3%)	258 (12.8%)	521 (14.5%)	297 (21.0%)	<0.001
	Statin	5454 (77.9%)	1614 (80.0%)	2819 (78.9%)0	1021 (72.3%)	<0.001

TG/HDL-C, triglyceride to high-density lipoprotein cholesterol; eGFR, estimated glomerular filtration rate; MI, myocardial infarction; STEMI, ST-segment elevation myocardial infarction; NSTEMI, non ST-segment elevation myocardial infarction; DM, diabetes mellitus; CAD, coronary artery disease; CK-MB, cratine kinase MB; cTnI, cardiac troponin I; HDL, high-density lipoprotein; LDL, low-density lipoprotein; hsCRP, high-sensitivity C-reaction protein; NT-proBNP, N-terminal prohormone of brain natriuretic peptide

^a^Comparison between patients with normal renal function, mild renal dysfunction, and moderate renal dysfunction.

**Fig 3:**
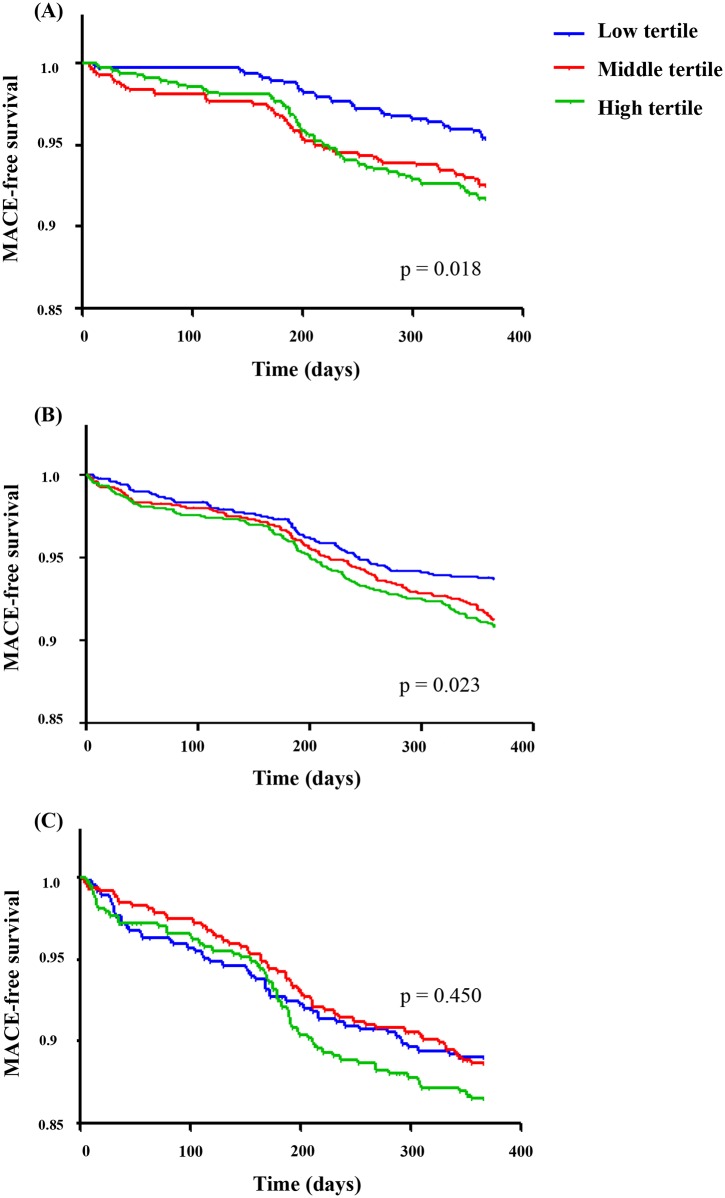
12-month MACE-free survival according to TG/HDL-C ratio in subgroup analyses. (A) Normal renal function (eGFR ≥ 90 mL/min/1.73m^2^). (B) Mild renal dysfunction (eGFR ≥ 60 to < 90 mL/min/1.73m^2^). (C) Moderate renal dysfunction (eGFR < 60 mL/min/1.73m^2^). MACEs, major adverse cardiovascular events; TG/HDL-C, triglyceride to high-density lipoprotein cholesterol; eGFR, estimated glomerular filtration rate

Fig 3 shows 12-month MACEs free survival across the TG/HDL-C ratio values in subgroup analyses according to renal function. The log-rank test revealed a significant association between the TG/HDL-C ratio and MACEs-free survival in the normal renal function and mild renal dysfunction groups (Fig 3A and 3B; p = 0.018, and p = 0.023; respectively). However, the higher TG/HDL-C ratio lost its predictive value in patients with moderate renal dysfunction (Fig 3C; p = 0.450).

The HRs given TG/HDL-C ratio tertile were analyzed using Cox regression analysis in a crude model, as well as in a model adjusted for age, gender, and traditional cardiovascular risk factors such as BMI, hypertension, DM, previous CAD, dyslipidemia, and current smoking. We also adjusted for medications that have been reported to be associated with lipid profile (Table 5). In the crude model, tertiles of TG/HDL-C ratio were significantly associated with increasing risk for 12-month MACEs in individuals with normal renal function and mild renal dysfunction (p = 0.006, and p = 0.009, respectively). However, in patients with moderate renal dysfunction, the TG/HDL-C ratio lost its predictive effect on 12-month MACEs (p = 0.262). The predictive value of TG/HDL-C ratio for 12-month MACEs remained similar pattern even when adjusting for covariates in the analyses.

**Table 5:** Cox regression for 12-month MACEs according to TG/HDL-C ratio.

			MACEs		
Model	eGFR (mL/min/1.73m^2^)	TG/HDL-C tertiles	Events (%)	HR (95% CI)	P value
Crude	≥ 90	Low tertile	31 (22.6%)	1	
		Middle tertile	50 (36.5%)	1.65 (1.05–2.59)	0.028
		High tertile	56 (40.9%)	1.84 (1.19–2.86)	0.006
	≥ 60 to < 90	Low tertile	75 (26%)	1	
		Middle tertile	104 (36%)	1.39 (1.04–1.88)	0.028
		High tertile	110 (38.1%)	1.47 (1.10–1.98)	0.009
	< 60	Low tertile	51 (30.5%)	1	
		Middle tertile	53 (31.7%)	1.02 (0.69-–1.50)	0.915
		High tertile	63 (37.7%)	1.24 (0.85–1.79)	0.262
Adjusted 1*	≥ 90	Low tertile	31 (22.6%)	1	
		Middle tertile	50 (36.5%)	1.68 (1.07–2.63)	0.023
		High tertile	56 (40.9%)	1.92 (1.23–3.01)	0.004
	≥ 60 to < 90	Low tertile	75 (26%)	1	
		Middle tertile	104 (36%)	1.42 (1.06–1.91)	0.020
		High tertile	110 (38.1%)	1.55 (1.15–2.09)	0.004
	< 60	Low tertile	51 (30.5%)	1	
		Middle tertile	53 (31.7%)	1.02 (0.69–1.50)	0.906
		High tertile	63 (37.7%)	1.25 (0.86–1.82)	0.245
Adjusted 2**	≥ 90	Low tertile	31 (22.6%)	1	
		Middle tertile	50 (36.5%)	1.58 (1.01–2.49)	0.049
		High tertile	56 (40.9%)	1.65 (1.04–2.60)	0.018
	≥ 60 to < 90	Low tertile	75 (26%)	1	
		Middle tertile	104 (36%)	1.51 (1.10–2.05)	0.010
		High tertile	110 (38.1%)	1.60 (1.16-–2.19)	0.004
	< 60	Low tertile	51 (30.5%)	1	
		Middle tertile	53 (31.7%)	1.03 (0.69–1.54)	0.873
		High tertile	63 (37.7%)	1.25 (0.84–1.86)	0.274
Adjusted 3***	≥ 90	Low tertile	31 (22.6%)	1	
		Middle tertile	50 (36.5%)	1.57 (0.99–2.48)	0.052
		High tertile	56 (40.9%)	1.64 (1.04–2.60)	0.035
	≥ 60 to < 90	Low tertile	75 (26%)	1	
		Middle tertile	104 (36%)	1.46 (1.08–1.98)	0.014
		High tertile	110 (38.1%)	1.56 (1.14–2.12)	0.005
	< 60	Low tertile	51 (30.5%)	1	
		Middle tertile	53 (31.7%)	1.03 (0.69–1.53)	0.905
		High tertile	63 (37.7%)	1.23 (0.82–1.83)	0.317

*Adjusted 1: Age, Gender;

**Adjusted 2: Adjusted 1 plus BMI, hypertension, DM, dyslipidemia, previous CAD, smoking,

***Adjusted 3: Adjusted 2 plus ACE inhibitors, Angiotensin-receptor blocker, and statin.

TG/HDL-C, triglyceride to high-density lipoprotein cholesterol; eGFR, estimated glomerular filtration rate; MACEs, major adverse cardiovascular events; BMI, body mass index; DM, diabetes mellitus; CAD, coronary artery disease

We also analyzed patients in according to subdivide renal function [normal renal function (eGFR ≥ 90 mL/min/1.73m^2^), mild renal dysfunction (eGFR ≥ 60 to < 90 mL/min/1.73m^2^), moderate renal dysfunction (eGFR ≥ 30 to < 60 mL/min/1.73m^2^), and severe renal dysfunction (eGFR < 30 mL/min/1.73m^2^)]. These analyses showed the same results (S1 Table).

## Discussion

To our knowledge, this is the first study to evaluate the predictive value of TG/HDL-C ratio in according to renal function. Our three principal findings are: 1) Higher TG/HDL-C ratio independently predicts 12-month MACEs in patients with AMI; 2) The TG/HDL-C ratio also showed a similar predictive effect on 12-month MACEs in patients with normal renal function (eGFR ≥ 90 mL/min/1.73m^2^) and mild renal dysfunction (eGFR ≥ 60 to < 90 mL/min/1.73m^2^); and 3) The TG/HDL-C ratio lost its predictive value in patients with moderate renal dysfunction (eGFR < 60 mL/min/1.73m^2^).

Since Gaziano et al. [14] first reported the predictive role of TG/HDL-C ratio for the risk of MI, several studies have also reported that this ratio is a powerful predictor of CVD risk and cardiovascular mortality [9] [15]. The followings are some suppositions for how TG/HDL-C ratio predicts worse cardiovascular outcomes. First, the TG/HDL-C ratio reflects the balance between atherogenic and protective lipoproteins. It also reflects the complex interactions involved in lipoprotein metabolism and can thus be useful in predicting plasma atherogenicity [16]. Second, inflammatory reactions may be another pathological mechanism. High TG and low HDL-C levels are strongly associated with the presence of small and dense LDL cholesterol particles [6]. These LDL particles are easily taken up by arterial tissue and are highly prone to oxidation. Accumulation of oxidized LDL cholesterol stimulates monocytes and macrophages to secrete proinflammatory cytokines and chemokines [17]. Lastly, insulin resistance may be a cause of pathology. The TG/HDL-C ratio is also known as an acknowledged marker of insulin resistance [18–20]. Razani et al. [21] reported that insulin resistance was an important contributor to the acceleration of atherogenesis.

The predictive role of the TG/HDL-C ratio has been validated previously not only in the general population but also in patients with several metabolic disorders. Turak et al. [3] reported that a high TG/HDL-C ratio is associated with MACEs and with total mortality in patients with essential hypertension. Wan et al. [7] showed that the TG/HDL-C ratio is an independent predictor of all-cause mortality even after adjusting for traditional risk factors in patients with acute coronary syndrome. Zoppini et al. [8] demonstrated that high TG/HDL-C ratio is a risk factor for cardiovascular and all-cause mortality in type 2 DM patients. There have also been a few studies which evaluated the prognostic impact of the TG/HDL-C ratio in patients with chronic kidney disease [4, 10]. However, these studies did not report changes in the predictive value of the TG/HDL-C ratio in according to renal function.

In this study, we showed that the TG/HDL-C ratio lost its prospective value in patients with moderate renal dysfunction. We could not determine causality or the underlying mechanisms for this phenomenon. However, we speculate that unlike patients with other disorders, patients with renal dysfunction have several non-traditional risk factors for CVD, such as anemia, alterations in calcium and phosphorus metabolism, and volume overload [22, 23]. Malnutrition can worsen cardiovascular outcomes by aggravating existing inflammation, accelerating atherosclerosis, and increasing susceptibility to infection [24]. Some studies have failed to establish a significant association between dyslipidemia and CVD risk in patients with renal dysfunction [25]. There have been some studies that reported an inverse relationship between conventional risk factors and mortality in renal dysfunction. This paradoxical phenomenon has been called “reverse epidemiology” [26, 27]. Kalantar-Zadeh et al. [26] described this phenomenon in patients with end stage renal disease (ESRD) who were on dialysis. Csaba et al. [27] showed that patients with chronic kidney disease (CKD) who are not yet on dialysis also show this phenomenon. There must be some prevailing factors that make CKD patients more susceptible to CVD.

Our study has several potential limitations which should be considered. First, the GFR was not measured directly. Rather, it was estimated using the MDRD study equation. This could have led to an over- or underestimation of renal function. Second, some patients may have been misclassified because the baseline lipid parameters and serum creatinine were only measured once. We might have missed changes in the laboratory results, and the impact of such changes on clinical outcome could not be taken into account. In Fig 3A, middle tertile group showed lower MACE-free survival rate in first 200 days compared to high tertile group. This cannot excluded the possibility of changing values of serum creatinine or TG/HDL-C ratio at this time point. Third, we did not have information on the dosages of statins, concomitant drugs that impact the serum lipid profile. Although we took these things into consideration during analyses, we were not able to get the information. Fourth, we did not include some factors that could influence the occurrence of MACEs. For instance, nocturnal hypertension has been reported as an independent risk factor for cardiovascular disease [28]. Unfortunately, we could not check 24-hour ambulatory blood pressure in this cohort study. Fifth, this was an observational study in a very specific population. There might be some differences in MACEs in our study population versus the general population. In particular, the clinical follow-up duration was short, and the in inclusion criteria could have contributed to such differences. Accordingly, the results should be generalized with caution. Finally, we did not assess long-term clinical outcomes. Prospective clinical studies with a longer follow-up duration are needed to define the clinical role of the TG/HDL-C ratio.

There are also some strengths to this study. First, it was a population-based study and that included a large number of patients. Second, we adjusted for various established risk factors and multiple confounders in the outcome analysis. Finally, to our knowledge, this is the first study to investigate the predictive value of the TG/HDL-C ratio in detail according to renal function.

In conclusion, in patients with AMI, the TG/HDL-C ratio is a useful independent predictor of 12-month MACEs. However, this ratio lost its predictive power in patients with moderate renal dysfunction. Further longitudinal and comparative studies are needed to investigate the mechanisms underlying this phenomenon.

## Appendix

Institutional Review Board of Participating Sites: The Korea Acute Myocardial Infarction Registry (KAMIR) was approved by the institutional review board (IRB) at each participating center (Kyunghee University IRB, Asan Medical Center IRB, Ajou University Hospital IRB, Busan Hanseo Hospital IRB, Busan National University Hospital IRB, Choongbook National University Hospital IRB, Chungnam National University Hospital IRB, Catholic University Hospital IRB, Chonbuk National University Hospital IRB, Chosun University Hospital IRB, Chung-Ang University Hospital IRB, Dankook University Hospital IRB, Daegu Catholic University Medical Center IRB, Dong-A University Medical Center IRB, Daejeon St. Mary's Hospital IRB, Dongguk University Gyeongju Hospital IRB, Gyeongsang National University Hospital IRB, Hallym University Kandong Sacred Heart Hospital IRB, Hallym University Sacred Heart Hospital IRB, Inje University Haeundae Paik hospital IRB, Inje University Sanggye Paik Hospital IRB, Inha University Hospital IRB, Jeju National University Hospital IRB, Kyungpook National University Hospital IRB, Keimyung University Hospital IRB, Korea University Guro Hospital IRB, Konyang University Hospital IRB, Kwangju Christian Hospital IRB, Maryknoll Medical Center IRB, National Health Insurance Corporation Ilsan Hospital IRB, Yeungnam University Hospital IRB, Yonsei University Severance Hospital IRB, Yonsei University Wonju Hospital IRB, Presbyterian Medical Center IRB, Seoul National University Hospital IRB, Seoul National University Bundang Hospital IRB, Soon Chun Hyang University Bucheon Hospital IRB, Soon Chun Hyang University Cheonan Hospital IRB, Samsung Medical Center IRB, Sun General Hospital IRB, Saint Carollo Hospital IRB, Wonkwang University Hospital IRB and Chonnam National University Hospital IRB).

## Supporting Information

S1 Table Cox regression for 12-month MACEs according to TG/HDL-C ratio in subdivided group. (DOCX)

## References

[1] Pagidipati NJ, Gaziano TA. Estimating deaths from cardiovascular disease: a review of global methodologies of mortality measurement. Circulation. 2013;127(6):749–56. 10.1161/CIRCULATIONAHA.112.128413 23401116 PMC3712514

[2] Lloyd-Jones DM. Cardiovascular risk prediction: basic concepts, current status, and future directions. Circulation. 2010;121(15):1768–77. 10.1161/CIRCULATIONAHA.109.849166 . 20404268

[3] Turak O, Afsar B, Ozcan F, Oksuz F, Mendi MA, Yayla C, et al The Role of Plasma Triglyceride/High-Density Lipoprotein Cholesterol Ratio to Predict New Cardiovascular Events in Essential Hypertensive Patients. Journal of clinical hypertension. 2015 10.1111/jch.12758 . 26694089 PMC8031528

[4] Sonmez A, Yilmaz MI, Saglam M, Unal HU, Gok M, Cetinkaya H, et al The role of plasma triglyceride/high-density lipoprotein cholesterol ratio to predict cardiovascular outcomes in chronic kidney disease. Lipids in health and disease. 2015;14:29 10.1186/s12944-015-0031-4 25885289 PMC4407467

[5] Dobiasova M, Frohlich J. The plasma parameter log (TG/HDL-C) as an atherogenic index: correlation with lipoprotein particle size and esterification rate in apoB-lipoprotein-depleted plasma (FER(HDL)). Clinical biochemistry. 2001;34(7):583–8. . 11738396 10.1016/s0009-9120(01)00263-6

[6] Kang HT, Shim JY, Lee YJ, Lee JE, Linton JA, Kim JK, et al Association between the ratio of triglycerides to high-density lipoprotein cholesterol and chronic kidney disease in Korean adults: the 2005 Korean National Health and Nutrition Examination Survey. Kidney & blood pressure research. 2011;34(3):173–9. 10.1159/000323895 . 21502765

[7] Wan K, Zhao J, Huang H, Zhang Q, Chen X, Zeng Z, et al The association between triglyceride/high-density lipoprotein cholesterol ratio and all-cause mortality in acute coronary syndrome after coronary revascularization. PloS one. 2015;10(4):e0123521 10.1371/journal.pone.0123521 25880982 PMC4399840

[8] Zoppini G, Targher G, Negri C, Stoico V, Gemma ML, Bonora E. Usefulness of the triglyceride to high-density lipoprotein cholesterol ratio for predicting mortality risk in type 2 diabetes: role of kidney dysfunction. Atherosclerosis. 2010;212(1):287–91. 10.1016/j.atherosclerosis.2010.04.035 . 20510417

[9] Bittner V, Johnson BD, Zineh I, Rogers WJ, Vido D, Marroquin OC, et al The triglyceride/high-density lipoprotein cholesterol ratio predicts all-cause mortality in women with suspected myocardial ischemia: a report from the Women's Ischemia Syndrome Evaluation (WISE). American heart journal. 2009;157(3):548–55. 10.1016/j.ahj.2008.11.014 19249427 PMC2677623

[10] Chen HY, Tsai WC, Chiu YL, Hsu SP, Pai MF, Yang JY, et al Triglyceride to high-density lipoprotein cholesterol ratio predicts cardiovascular outcomes in prevalent dialysis patients. Medicine. 2015;94(10):e619 10.1097/MD.0000000000000619 25761189 PMC4602469

[11] von Elm E, Altman DG, Egger M, Pocock SJ, Gotzsche PC, Vandenbroucke JP, et al The Strengthening the Reporting of Observational Studies in Epidemiology (STROBE) statement: guidelines for reporting observational studies. Lancet. 2007;370(9596):1453–7. 10.1016/S0140-6736(07)61602-X . 18064739

[12] Levey AS, Bosch JP, Lewis JB, Greene T, Rogers N, Roth D. A more accurate method to estimate glomerular filtration rate from serum creatinine: a new prediction equation. Modification of Diet in Renal Disease Study Group. Annals of internal medicine. 1999;130(6):461–70. . 10075613 10.7326/0003-4819-130-6-199903160-00002

[13] Bakris GL, Smith AC, Richardson DJ, Hung E, Preston R, Goldberg R, et al Impact of an ACE inhibitor and calcium antagonist on microalbuminuria and lipid subfractions in type 2 diabetes: a randomised, multi-centre pilot study. J Hum Hypertens. 2002;16(3):185–91. 10.1038/sj.jhh.1001315 . 11896508

[14] Gaziano JM, Hennekens CH, O'Donnell CJ, Breslow JL, Buring JE. Fasting triglycerides, high-density lipoprotein, and risk of myocardial infarction. Circulation. 1997;96(8):2520–5. . 9355888 10.1161/01.cir.96.8.2520

[15] Jeppesen J, Hein HO, Suadicani P, Gyntelberg F. Low triglycerides-high high-density lipoprotein cholesterol and risk of ischemic heart disease. Archives of internal medicine. 2001;161(3):361–6. . 11176761 10.1001/archinte.161.3.361

[16] Millan J, Pinto X, Munoz A, Zuniga M, Rubies-Prat J, Pallardo LF, et al Lipoprotein ratios: Physiological significance and clinical usefulness in cardiovascular prevention. Vasc Health Risk Manag. 2009;5:757–65. 19774217 PMC2747394

[17] Glass CK, Witztum JL. Atherosclerosis. the road ahead. Cell. 2001;104(4):503–16. . 11239408 10.1016/s0092-8674(01)00238-0

[18] Hadaegh F, Hatami M, Tohidi M, Sarbakhsh P, Saadat N, Azizi F. Lipid ratios and appropriate cut off values for prediction of diabetes: a cohort of Iranian men and women. Lipids in health and disease. 2010;9:85 10.1186/1476-511X-9-85 20712907 PMC2933665

[19] Kim JS, Kang HT, Shim JY, Lee HR. The association between the triglyceride to high-density lipoprotein cholesterol ratio with insulin resistance (HOMA-IR) in the general Korean population: based on the National Health and Nutrition Examination Survey in 2007–2009. Diabetes Res Clin Pract. 2012;97(1):132–8. 10.1016/j.diabres.2012.04.022 . 22607906

[20] Ren X, Chen ZA, Zheng S, Han T, Li Y, Liu W, et al Association between Triglyceride to HDL-C Ratio (TG/HDL-C) and Insulin Resistance in Chinese Patients with Newly Diagnosed Type 2 Diabetes Mellitus. PloS one. 2016;11(4):e0154345 10.1371/journal.pone.0154345 27115999 PMC4846162

[21] Razani B, Chakravarthy MV, Semenkovich CF. Insulin resistance and atherosclerosis. Endocrinol Metab Clin North Am. 2008;37(3):603–21, viii 10.1016/j.ecl.2008.05.001 18775354 PMC2639785

[22] Cheung AK, Sarnak MJ, Yan G, Dwyer JT, Heyka RJ, Rocco MV, et al Atherosclerotic cardiovascular disease risks in chronic hemodialysis patients. Kidney Int. 2000;58(1):353–62. 10.1046/j.1523-1755.2000.00173.x . 10886582

[23] Sarnak MJ, Levey AS. Cardiovascular disease and chronic renal disease: a new paradigm. Am J Kidney Dis. 2000;35(4 Suppl 1):S117–31. . 10766010 10.1016/s0272-6386(00)70239-3

[24] Pecoits-Filho R, Lindholm B, Stenvinkel P. The malnutrition, inflammation, and atherosclerosis (MIA) syndrome—the heart of the matter. Nephrol Dial Transplant. 2002;17 Suppl 11:28–31. . 12386254 10.1093/ndt/17.suppl_11.28

[25] Tsimihodimos V, Mitrogianni Z, Elisaf M. Dyslipidemia associated with chronic kidney disease. Open Cardiovasc Med J. 2011;5:41–8. 10.2174/1874192401105010041 21643500 PMC3106357

[26] Kalantar-Zadeh K, Block G, Humphreys MH, Kopple JD. Reverse epidemiology of cardiovascular risk factors in maintenance dialysis patients. Kidney Int. 2003;63(3):793–808. 10.1046/j.1523-1755.2003.00803.x . 12631061

[27] Kovesdy CP, Anderson JE. Reverse epidemiology in patients with chronic kidney disease who are not yet on dialysis. Seminars in dialysis. 2007;20(6):566–9. 10.1111/j.1525-139X.2007.00335.x . 17991206

[28] Unal HU, Basaran Y, Gezer M. The Triglyceride to High-Density Lipoprotein Cholesterol Ratio Is a Useful Marker to Predict Unfavorable Cardiovascular Outcomes, But Other Confounding Factors Should Be Considered. Journal of clinical hypertension. 2016 10.1111/jch.12807 . 26951914 PMC8032185

