# Supplementary material for: The Predictive Role of Serum Triglyceride to High-Density Lipoprotein Cholesterol Ratio According to Renal Function in Patients with Acute Myocardial Infarction
Source: PLoS One. 2016 Oct 27;11(10):e0165484. doi: 10.1371/journal.pone.0165484 (PMC5082929; doi:10.1371/journal.pone.0165484)
Supplement: S1 Table — (DOCX) [file pone.0165484.s001.docx]

**Supplemental table 1. Cox regression for 12-month MACEs according to TG/HDL-C ratio in subdivided group.**

|  |  |  | MACEs | | |
| --- | --- | --- | --- | --- | --- |
| Model | eGFR (mL/min/1.73m^2^) | TG/HDL-C tertiles | Events (%) | HR (95% CI) | P value^a^ |
| Crude | ≥ 90 | Low tertile | 31 (22.6%) | 1 |  |
|  |  | Middle tertile | 50 (36.5%) | 1.65 (1.05–2.59) | 0.028 |
|  |  | High tertile | 56 (40.9%) | 1.84 (1.19–2.86) | 0.006 |
|  | ≥ 60 to < 90 | Low tertile | 75 (26%) | 1 |  |
|  |  | Middle tertile | 104 (36%) | 1.39 (1.04–1.88) | 0.028 |
|  |  | High tertile | 110 (38.1%) | 1.47 (1.10–1.98) | 0.009 |
|  | ≥ 30 to < 60 | Low tertile | 51 (10.9%) | 1 |  |
|  |  | Middle tertile | 53 (11.2%) | 1.02 (0.69–1.50) | 0.915 |
|  |  | High tertile | 63 (13.3%) | 1.24 (0.85–1.79) | 0.262 |
|  | < 30 | Low tertile | 10 (14.5%) | 1 |  |
|  |  | Middle tertile | 7 (10.3%) | 0.71 (0.27–1.85) | 0.477 |
|  |  | High tertile | 11 (16.4%) | 1.17 (0.49–2.75) | 0.722 |
| Adjusted 1* | ≥ 90 | Low tertile | 31 (22.6%) | 1 |  |
|  |  | Middle tertile | 50 (36.5%) | 1.68 (1.07–2.63) | 0.023 |
|  |  | High tertile | 56 (40.9%) | 1.92 (1.23–3.01) | 0.004 |
|  | ≥ 60 to < 90 | Low tertile | 75 (26%) | 1 |  |
|  |  | Middle tertile | 104 (36%) | 1.42 (1.06–1.91) | 0.020 |
|  |  | High tertile | 110 (38.1%) | 1.55 (1.15–2.09) | 0.004 |
|  | ≥ 30 to < 60 | Low tertile | 51 (10.9%) | 1 |  |
|  |  | Middle tertile | 53 (11.2%) | 1.02 (0.69–1.50) | 0.906 |
|  |  | High tertile | 63 (13.3%) | 1.25 (0.86–1.82) | 0.245 |
|  | < 30 | Low tertile | 10 (14.5%) | 1 |  |
|  |  | Middle tertile | 7 (10.3%) | 0.73 (0.28–1.92) | 0.422 |
|  |  | High tertile | 11 (16.4%) | 1.39 (0.57–3.40) | 0.461 |
| Adjusted 2** | ≥ 90 | Low tertile | 31 (22.6%) | 1 |  |
|  |  | Middle tertile | 50 (36.5%) | 1.58 (1.01–2.49) | 0.049 |
|  |  | High tertile | 56 (40.9%) | 1.65 (1.04–2.60) | 0.018 |
|  | ≥ 60 to < 90 | Low tertile | 75 (26%) | 1 |  |
|  |  | Middle tertile | 104 (36%) | 1.51 (1.10–2.05) | 0.010 |
|  |  | High tertile | 110 (38.1%) | 1.60 (1.16–2.19) | 0.004 |
|  | ≥ 30 to < 60 | Low tertile | 51 (30.5%) | 1 |  |
|  |  | Middle tertile | 53 (31.7%) | 1.04 (0.69–1.55) | 0.860 |
|  |  | High tertile | 63 (37.7%) | 1.24 (0.84–1.85) | 0.283 |
|  | < 30 | Low tertile | 10 (14.5%) | 1 |  |
|  |  | Middle tertile | 7 (10.3%) | 0.78 (0.37–2.29) | 0.655 |
|  |  | High tertile | 11 (16.4%) | 1.07 (0.38–3.05) | 0.897 |
| Adjusted 3** | ≥ 90 | Low tertile | 31 (22.6%) | 1 |  |
|  |  | Middle tertile | 50 (36.5%) | 1.58 (1.01–2.49) | 0.049 |
|  |  | High tertile | 56 (40.9%) | 1.65 (1.04–2.60) | 0.018 |
|  | ≥ 60 to < 90 | Low tertile | 75 (26%) | 1 |  |
|  |  | Middle tertile | 104 (36%) | 1.51 (1.10–2.05) | 0.010 |
|  |  | High tertile | 110 (38.1%) | 1.60 (1.16–2.19) | 0.004 |
|  | ≥ 30 to < 60 | Low tertile | 51 (30.5%) | 1 |  |
|  |  | Middle tertile | 53 (31.7%) | 1.03 (0.69–1.23) | 0.905 |
|  |  | High tertile | 63 (37.7%) | 1.23 (0.82–1.83) | 0.317 |
|  | < 30 | Low tertile | 10 (14.5%) | 1 |  |
|  |  | Middle tertile | 7 (10.3%) | 0.77 (0.26–2.29) | 0.638 |
|  |  | High tertile | 11 (16.4%) | 1.13 (0.39–3.24) | 0.825 |

*Adjusted 1: Age and Gender; **Adjusted 2: Adjusted 1 plus BMI, hypertension, DM, dyslipidemia, previous CAD, and smoking; ***Adjusted 3: Adjusted 2 plus ACE inhibitors, Angiotensin-receptor blocker, and statin.TG/HDL-C, triglyceride to high-density lipoprotein cholesterol; eGFR, estimated glomerular filtration rate; MACEs, major adverse cardiovascular events; BMI, body mass index; DM, diabetes mellitus; CAD, coronary artery disease

^a^Comparison between patients with low, middle, and high tertile of TG/HDL-C ratio.
